# Multivalent pIX phage display selects for distinct and improved antibody properties

**DOI:** 10.1038/srep39066

**Published:** 2016-12-14

**Authors:** Lene S. Høydahl, Nicolay R. Nilssen, Kristin S. Gunnarsen, M. Fleur du Pré, Rasmus Iversen, Norbert Roos, Xi Chen, Terje E. Michaelsen, Ludvig M. Sollid, Inger Sandlie, Geir Å. Løset

**Affiliations:** 1 Centre for Immune Regulation and Department of Immunology, University of Oslo and Oslo University Hospital, N-0372 Oslo, Norway; 2 Department of Biosciences, University of Oslo, N-0316 Oslo, Norway; 3 Department of Immunology, Norwegian Institute of Public Health, N-0403 Oslo, Norway; 4 School of Pharmacy, University of Oslo, N-0316 Oslo, Norway; 5 KG Jebsen Coeliac Disease Research Centre and Department of Immunology, University of Oslo, N-0372 Oslo, Norway; 6 Nextera AS, N-0349 Oslo, Norway

## Abstract

Phage display screening readily allows for the identification of a multitude of antibody specificities, but to identify optimal lead candidates remains a challenge. Here, we direct the antibody-capsid fusion away from the signal sequence-dependent secretory SEC pathway in *E. coli* by utilizing the intrinsic signal sequence-independent property of pIX to obtain virion integration. This approach was combined with the use of an engineered helper phage known to improve antibody pIX display and retrieval. By direct comparison with pIII display, we demonstrate that antibody display using this pIX system translates into substantially improved retrieval of desired specificities with favorable biophysical properties in *de novo* selection. We show that the effect was due to less *E. coli* host toxicity during phage propagation conferred by the lack of a signal sequence. This pIX combinatorial display platform provides a generic alternative route for obtaining good binders with high stability and may thus find broad applicability.

Phage display technology has been extensively modified and improved since its introduction three decades ago and is still the most important molecular evolution technology available^1^. In particular, the use of phage display in antibody discovery has provided high-quality affinity reagents for use in research, proteome-scale experiments and therapeutic intervention^2^. The quality of the antibody library is crucial for successful antibody discovery. Thus, attention has been focused on library size and design of the displayed antibody format^3^. In comparison, less is known about how the choice of capsid protein used for display influences library performance. In the current study, we have assessed such differences between pIII and pIX as display scaffolds to gain insight into how the choice of capsid protein influences the characteristics of the selected clones.

Polypeptides of interest have been displayed on all five phage structural proteins, but pIII is by far the dominating display capsid used in antibody display and selection^4^. However, the use of pIII is associated with well-documented negative effects on selection performance, such as clone-dependent reduced phage infectivity and propagation, which cause repertoire bias^5^. In addition, preferential amplification of non-functional or truncated clones in *E. coli* may impede successful selection of functional clones^6^,^7^,^8^. Thus, although pIII display technology is well established and has generated a wide range of binders, its limitations motivate search for further improvements.

Several reports have explored pIX as an alternative antibody display scaffold^9^,^10^,^11^. pIX and pIII are located at opposite tips on the virion, and the two proteins differ in their mechanisms of translocation to the *E. coli* inner membrane prior to phage assembly. Whereas pIII is synthesized as a precursor with a post-translationally processed signal sequence and targets the SEC translocation pathway^12^, pIX altogether lacks a signal sequence, does not undergo post-translational processing and appears to depend on YidC for periplasmic targeting^12^,^13^. Early studies showed that an *N*-terminal GST fusion prevented pIX display, as pIX was found aggregated in the cytosol of the *E. coli* host^14^. Therefore, ompA and pelB signal sequences were added with the aim to facilitate antibody display^15^, but later reports are conflicting with respect to how well such pIX display performs in comparison to display on pIII^9^,^16^,^17^. We have developed a display system, where pIX is used as display capsid without addition of a signal sequence to the fusion protein^18^,^19^.

In this study, we have for the first time tested the performance of this signal sequence-independent pIX display in diverse antibody library selection and compare with conventional SEC-dependent pIII display. We compared the two strategies in independent side-by-side selections using both immune and naïve scFv libraries. In addition, we compared the contribution of display valence on the outcome. Our results highlight two major novel findings. First, the hit-rate of specific clones was higher for pIX display. In the first selection, only pIX display retrieved specific binders of good affinity. In the second selection, different repertoires were retrieved in a capsid-dependent manner, allowing for a comparison, and the pIX-selected clones had the higher thermostability and affinity. Second, we demonstrate that multivalent display is a prerequisite for the favorable performance of pIX.

## Results

### Selection and characterization of an antibody directed towards a celiac disease patient-derived anti-TG2 monoclonal antibody

**Figure 1:**
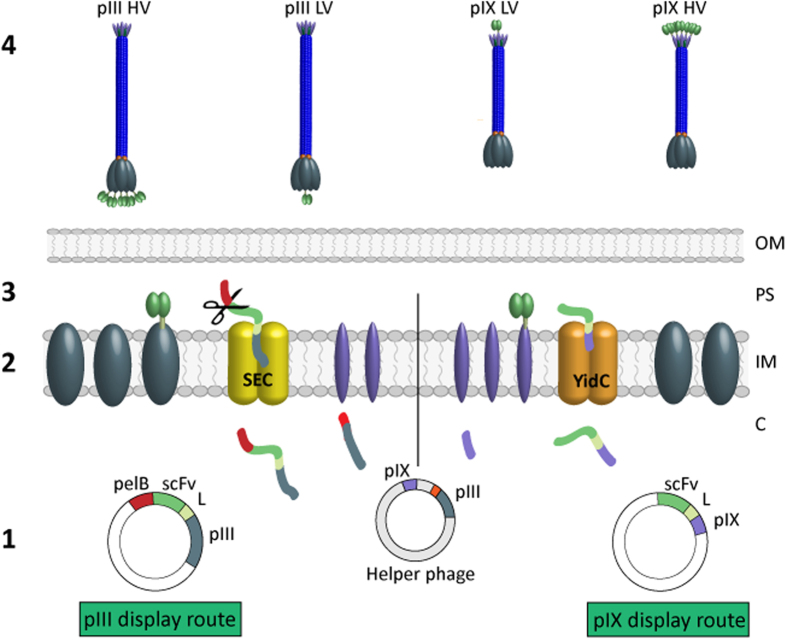
Overview of pIII and pIX display routes and the four library groups. (**1**) Schematic illustration of pIII and pIX phagemids. The scFv-pIII contains an *N*-terminal pelB signal sequence, while pIX does not. The scFvs are connected to the capsid proteins by a linker (L). A helper phage encodes wt pIII and wt pIX, resulting in LV display as shown. By using modified helper phages that do not encode either wt capsid protein, the sole source of the capsid protein is the scFv-capsid fusion encoded by the phagemids, resulting in HV display of the fusion. (**2**) pIII and pIX use different translocation mechanisms, SEC and YidC, respectively, for transport from the cytosol (C) to the periplasmic space (PS). Both capsid proteins are integral inner membrane (IM) proteins prior to virion assembly. (**3**) Post-translational leader processing cleaves the leader on pIII and scFv-pIII in the PS. (**4**) The virions are released through the outer membrane (OM), resulting in the four libraries.

**Figure 2:**
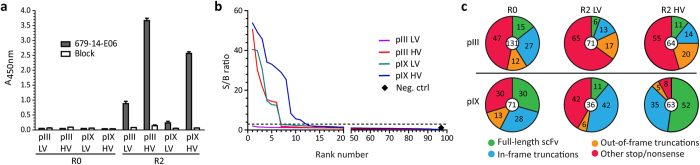
Polyclonal phage ELISA, single-clone screening for mAb 679-14-E06-reactivity and single-clone sequences. (**a**) Normalized phage pools from R0 and R2 were analyzed for the presence of binders to the target mAb by polyclonal phage ELISA. The results are given as mean ± SD of duplicates. The experiment was repeated independently giving the same results. (**b**) Single colonies from R2 were randomly picked for small-scale phagemid rescue and analyzed for target reactivity by ELISA (n = 2). All clones were rescued to HV exploiting functional affinity effects to maximize detection sensitivity. Hence, the LV and HV annotations refer to the valence during selection. A signal/background (S/B) ratio ≥3 was scored as specific (dotted line). Supernatant from empty *E. coli* TOP10F was included as a control. (**c**) Random colonies were picked for sequencing from R0 and after R2, and the sequence outputs were compared. The clones from R0 originate from the *E. coli* transformation outputs, i.e., before separation into LV and HV. Percent sequences within each category and the total number of sequences are indicated in the pie charts.

We aimed to select an antibody recognizing the V_H_/V_L_ combination of the monoclonal antibody (mAb) 679-14-E06 specific for the human self-antigen transglutaminase 2 (TG2)^20^. Murine scFv antibody libraries were assembled in parallel on pIII and pIX^5^ employing the identical PCR amplified V gene repertoire derived from mice that had been immunized with 679-14-E06 Fab (Supplementary Fig. S1). The libraries were packaged with helper phages directing either low valence (LV) or high valence (HV) display on pIII^21^ or pIX^19^, thus enabling direct comparison of how the capsid proteins and display valence influence the outcome of selection (overview of display routes and libraries are shown in Fig. 1). Selection of binders specific towards the V_H_/V_L_ combination was achieved by negative selection on a mixture of two 679-14-E06-related anti-TG2 mAbs that share either the target IGHV5-51 or IGKV1-5 gene segments, followed by positive selection on the target mAb. The round 2 (R2) outputs from all four selections were analyzed in a polyclonal phage ELISA to assess specific enrichment of binders (Fig. 2a). We observed a strong increase in reactivity towards mAb 679-14-E06 from both HV selections, an intermediate increase for pIII LV, while the pIX LV output showed only a slight increase in target reactivity. Rescue to HV forces all fusion-capsid proteins to display scFv and increases antigen reactivity compared to the LV counterparts (Supplementary Fig. S2). Single phage clones were then expanded and tested for specific target binding in phage ELISA (Fig. 2b). The R2 output from pIX HV contained the highest number, 12/95, of positive clones followed by pIII HV and pIX LV with 6 each. We found no binders in pIII LV. With one exception, all clones selected from the pIX libraries exhibited stronger target reactivity than those from the pIII libraries (Fig. 2b). Sequencing revealed that a single clone (denoted 2G9) dominated the R2 outputs from both pIX libraries (Supplementary Fig. S1). This clone was reformatted to full-length human IgG1 and confirmed to bind the target mAb by SPR (K_d_ ~137 nM) and to engineered A20 B cells expressing 679-14-E06 as B cell receptor (EC_50_ ~0.3 nM) (Supplementary Fig. S1). No binding towards the single V_H_ or V_L_-matched control mAbs was observed (Supplementary Fig. S1).

### HV display on pIX selects full-length and functional ORFs

The result from the single-clone phage screen prompted us to elucidate the differences in pIII and pIX selection dynamics by sequencing a number of random clones before (R0) and after (R2) selection (Fig. 2c). Both R0 libraries had a high content of non-functional clones with V_H_ truncations or frameshift mutations, partly caused by the library cloning procedure. Furthermore, during affinity selection, both the pIII LV and HV outputs exhibited a reduction in functional open reading frames (ORFs), as well as enrichment of clones with out-of-frame truncations and stop codons. The pIX LV output showed a similar trend, while the pIX HV output showed a strong enrichment of full-length, functional clones.

Sequencing of the pIII HV R2 output and phage ELISA on target mAb and the mAb sharing IGKV1-5, revealed that the clones with in-frame truncations were IGKV1-5-specific and enriched as binders after incomplete negative selection (Supplementary Fig. S1).

### *E. coli* host interference is more severe for pIII than for pIX fusions

**Figure 3:**
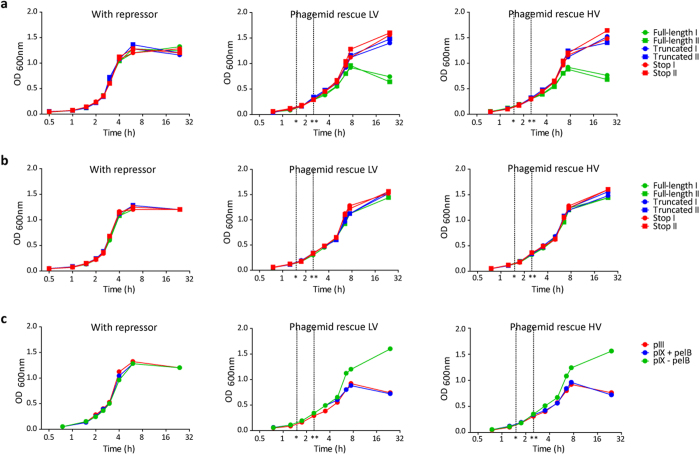
Effect of scFv capsid fusion expression and translocation mechanism on *E. coli* growth. (**a**,**b**) Two random, independent clones each representing full-length scFv, or scFv containing in-frame truncations, or stop codons displayed on either (**a**) pIII or (**b**) pIX were grown in the presence of glucose repressor to shut down scFv expression, or during LV and HV phage packaging as indicated. (**c**) A model scFv clone was displayed on pIII with pelB or on pIX with or without pelB, and *E. coli* growth was monitored in the presence of glucose, or during LV and HV phage packaging as indicated. (*) super infection, (**) medium exchange. Results are representative of three independent experiments.

The staggering differences in repertoire dynamics between the different display strategies observed during selection prompted us to generate host cell growth curves to evaluate how expression of in-frame truncated, non-sense or full-length scFvs on the two capsids affected *E. coli* propagation. We observed no differences in growth rate upon phagemid propagation in the presence of glucose repressor (Fig. 3a and b left panels). However, upon removal of the repressor to allow heterologous protein expression and phagemid packaging, full-length scFv on pIII caused growth retardation, as seen by the collapse in cell growth in mid-log phase (Fig. 3a middle and right panels). In contrast, we did not observe growth retardation with fusions displayed on pIX (Fig. 3b middle and right panels). The effect was independent of display valence. We reasoned that this counter-selection might be attributed either to inherent differences between the pIII and pIX capsids, or the different pathways used for translocation from the cytosol to the periplasmic space. To distinguish between the two and further elucidate the observed differences, we used a well-characterized mouse hybridoma-derived anti-NIP scFv^18^ in three different settings; standard pIII display (with pelB), display on pIX with pelB added *N*-terminally on the scFv, and display on pIX without pelB *N*-terminally on the scFv, and repeated the growth experiments. Again, during protein expression and phagemid packaging, the growth of host cell cultures with phagemids encoding pIII or pIX fusions with pelB signal sequence were greatly inhibited, while the presence of phagemids encoding pIX fusions devoid of signal sequence did not affect the host cells (Fig. 3c). Thus, the results strongly indicate that it was the presence of the pelB signal sequence on the scFv, and hence the SEC transport route from the cytosol to the periplasm, that was the major cause of the observed growth interference. Therefore, during library amplification between each selection round, clones with a growth advantage were preferentially amplified and outcompeted the full-length clones. In the mAb 679-14-E06 selection, the only group that effectively alleviated this effect was the pIX HV further pointing to the benefit of ORF selection (Fig. 2c).

### Parallel pIII and pIX selection from a naïve human scFv phage library

**Figure 4:**
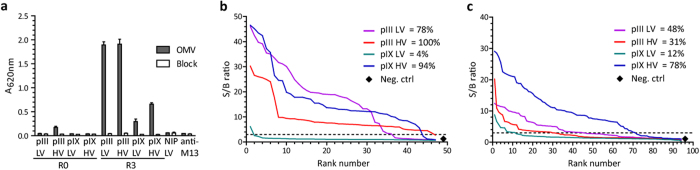
Polyclonal phage ELISA and single-clone screening for OMV-reactivity. (**a**) Normalized phage samples from R0 and R3 outputs were analyzed for binding to OMV by ELISA. Phages displaying an irrelevant specificity (scFv anti-NIP) were included as control. The experiment was repeated independently giving the same results. The results are given as mean ± SD of duplicates. (**b**) Random single colonies after R3 were rescued to HV display for all libraries. Samples were analyzed for OMV reactivity by ELISA and scored positive with a S/B ratio ≥3. The percentage of OMV positive clones within each library group is indicated. Supernatant from empty *E. coli* SS320 was included as a control. (**c**) The phage libraries after R3 were reformatted by batch-cloning for soluble scFv *E. coli* expression and random single clones analyzed for OMV binding by ELISA. A representative ELISA is shown (n = 2).

To investigate the generality of the apparent favorable selection performance of pIX display, we reformatted a previously described diverse and high quality protein L-purified human naïve scFv phage library^22^ to comparable pIII and pIX phagemid vectors^18^ (Supplementary Fig. S3). We then performed three rounds of selection on the OMV antigen of *N. meningitidis,* using the same four library groups as before, namely pIII and pIX both at either LV or at HV. Polyclonal phage ELISA of the selection outputs revealed OMV-specific reactivity in all four R3 outputs, with pIII outputs exhibiting a stronger reactivity than the pIX outputs (Fig. 4a). The pIII HV R0 output also bound weakly to the antigen before selection. To compare the outputs at the single-clone level, we picked random clones and screened for OMV reactivity in phage ELISA. The number of binders ranged from 4% to 100% of the clones tested (Fig. 4b). As previously reported, HV display during selection improved the retrieval of binders both from the pIII and the pIX libraries^19^,^21^, but this effect was more pronounced for pIX. Moreover, for pIX, HV display strongly increased target reactivity. By contrast, the positive pIII LV clones exhibited higher reactivity than the pIII HV clones. Thus, we found the highest single-clone reactivity in the pIX HV and pIII LV outputs.

### Display valence and choice of helper phage affect selection efficiency

**Figure 5:**
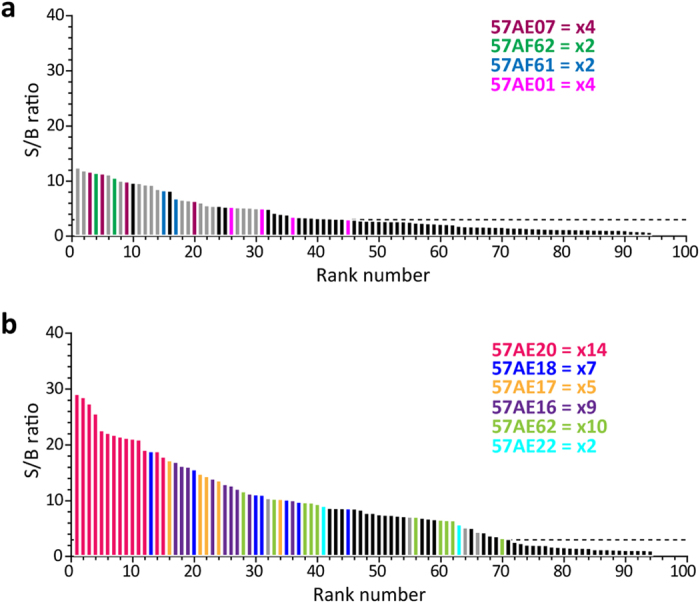
Distribution of enriched clones in pIII LV and pIX HV. (**a**,**b**) Modified from Fig. 4c to illustrate positions of enriched clones in (**a**) pIII LV and (**b**) pIX HV. Unique clones are identified by color and clone names from Supplementary Table S1. Grey bars indicate single clones (occurring once) within each library and black bars represent clones which are not sequence identified.

We then reformatted the selection outputs for soluble scFv expression, expanded random clones and tested for OMV reactivity in ELISA. In line with previous experience^23^,^24^, we observed a reduction in number of positive clones when reformatting from phage displayed to soluble scFv, except for the clones derived from the pIX LV selection (Fig. 4c). The reduction was less pronounced for pIX HV than for both pIII outputs. Comparing pIII LV and pIX HV, target reactivity was on average 2.3-fold higher in favor of the pIX-derived clones (Fig. 4c). Sequence analysis showed that clones enriched in the pIX HV selection occupied the highest ranked S/B positions, and that the most highly enriched clone (14 of 96 samples screened) occupied the 12 top S/B ranks (Fig. 5b). This contrasts the pIII LV route, where the few enriched clones were found scattered among unique clones and all these clones exhibited substantially lower S/B levels than what was seen for the pIX-derived clones (Fig. 5a).

The large reduction in target-reactive clones in pIII HV (from 100 to 31%) upon soluble scFv reformatting and the rather low target reactivity could be due to low intrinsic affinity or lack of scFv expression. Thus, we analyzed 10 representative clones from the pIII HV selection for protein expression level in addition to target binding. Here, only 4/10 scored as positive in OMV-binding, but all 10 expressed soluble, full-length protein at similar levels, and all appeared functionally folded, as they reacted with the conformation-dependent protein L (Supplementary Fig. S4). Sequence analysis revealed 7/10 clones to be identical (Supplementary Fig. S4), and this dominant clone was below detection threshold in 5/7 cases. In summary, the results support the notion that pIII HV display during selection increases the number of retrieved binders, but at the cost of reduced affinity^25^.

**Figure 6:**
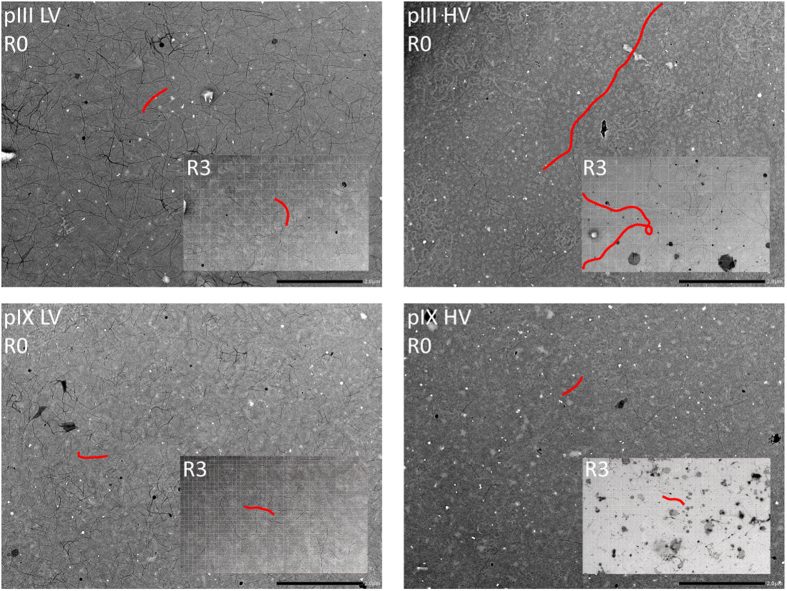
Transmission electron micrographs illustrating virion length. Phage samples from R0 and R3 were visualized by negative stain. Library samples were prepared and analyzed on two separate occasions. A representative virion in each library is highlighted in red. Scale bar 2 μm.

Morphological variations of the phages, such as presence of polyphages, could also contribute to the detection of weak binders in phage ELISA. Thus, we visualized phages from the libraries by transmission electron microscopy (TEM). Here, the pIII HV library stood out by containing a large fraction of polyphage, whereas this was not seen for the other three libraries (Fig. 6). Therefore, the weak target reactivity seen in pIII HV R0 prior to selection, as well as the very strong R3 reactivity (Fig. 4a), is likely due to signal amplification caused by very large phage particles. The absence of polyphages in pIII LV indicates that the phenomenon is not associated with pIII display itself, but rather related to the helper phage used to achieve HV display. Moreover, none of the pIX libraries contained polyphages. Thus, this virion morphology is not associated with HV display in general.

### Different repertoires are selected depending on display scaffold and valence

**Figure 7:**
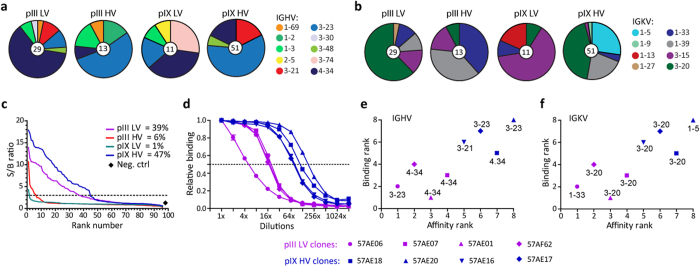
Sequence analysis and biophysical properties of the selected clones. (**a**,**b**) Random clones scored as OMV-specific binders in the soluble scFv screen were sequenced and grouped by (**a**) IGHV and (**b**) IGKV usage according to the IMGT database. (**c**) Combined medium and periplasmic fractions containing soluble scFv clones were challenged by heating 10 min at 55 °C before analysis of retained OMV binding. (**d**) Two-fold dilution series of periplasmic fractions were assessed for OMV binding by ELISA (n = 3). The curves were normalized to a relative binding of 1 for the highest concentration. Half of maximum binding is illustrated with a dotted line. The results are given as mean ± SD of duplicates. (**e**,**f**) Affinity rank versus binding rank illustrating how (**e**) IGHV and (**f**) IGKV usage correlate with the properties of the clones. The affinity rank of the clones was derived from (**d**) based on half maximum antigen binding. The binding rank was derived from Fig. 5a and b by calculating the average S/B ratio of the clones.

All libraries tested were based on the same V gene repertoire, which allowed an assessment of how capsid and display valence influenced V gene usage in the selected OMV-reactive clones. By sequencing clones identified in the soluble screen, we found the two LV outputs and the two HV outputs to be similar regarding IGHV usage, with preferential selection of IGHV4-34 in the LV and IGHV3-23 in the HV outputs, respectively (Fig. 7a). Strikingly, the IGKV usage in both the pIII LV and the pIX HV outputs was skewed towards a single gene segment, IGKV3-20. pIX HV also selected IGKV1-5, which was not found in the output from any other library (Fig. 7b). Sequence analysis further revealed that pIII LV selected a more diverse repertoire than pIX HV, with 21 compared to 10 unique sequences identified (Supplementary Table S1). However, this observation must be seen in context with the level of enrichment, which inevitably will affect the mere number of clones occupying the top hierarchy of identified binders in a screening, and here only 4/21 clones occurred more than once for pIII LV. On the other hand, pIX HV displayed a faster and stronger enrichment, as 6/10 clones occurred repeatedly and several were highly enriched. Importantly, the clones enriched via the pIX HV route predominantly used IGHV3, whereas clones enriched in pIII LV were dominated by IGHV4 (Fig. 7a).

To investigate differences in the biophysical properties of the selected repertoires, periplasmic samples of the same soluble scFv clones used in the screen were heat challenged at 55 °C for 10 min, followed by assessment of antigen binding (Fig. 7c, Supplementary Fig. S5 and Supplementary Table S1). Clones derived from pIX HV selection retained the highest percentage of positive clones, 47%, whereas 39% of pIII LV-derived clones were positive after heating. Samples derived from the two other libraries, pIII HV and pIX LV, only retained 6% and 1% positive clones, respectively. Finally, to get a more comprehensive picture of potential clonal differences in binding affinity, we tested the most highly enriched clones from pIX HV and pIII LV, including a unique pIII LV clone also identified in pIII HV, in serial dilution going from saturating to negligible OMV binding. In agreement with our clone screening results, all 4 clones from pIX HV showed the strongest target reactivity in a concentration-dependent manner strongly eluding to higher intrinsic affinity than the clones from pIII LV (Fig. 7d–f). Notably, the only clone (57AE06) shared between the pIII LV and HV groups exhibited the poorest target reactivity of all the tested clones.

In summary, affinity selection of the identical scFv pool using different display scaffolds translated into retrieval of distinct and non-overlapping clonal repertoires, and pIX HV-derived clones had superior biophysical properties with respect to both thermal resistance and target reactivity, when compared to the best performing pIII-derived clones, which all were from the LV group.

### pIX HV clones have superior antigen binding characteristics

**Figure 8:**
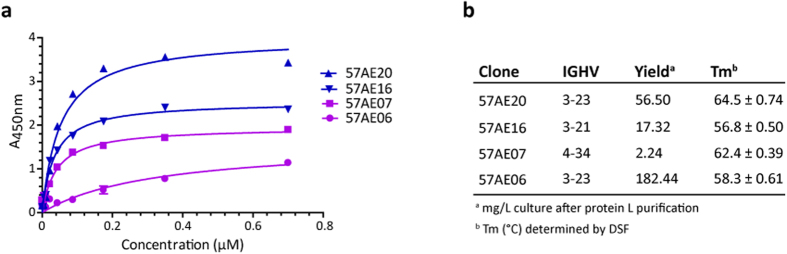
Extended biophysical assessment of affinity purified OMV-reactive scFv clones. (**a**) Representative saturation binding curves using normalized, titrated amounts of OMV-reactive scFv clones. The experiment was conducted with separately expressed samples; either protein L affinity purified scFv clones (n = 3) or scFv clones purified by protein L followed by size exclusion chromatography (n = 2) giving essentially the same result. The results are given as mean ± SD of duplicates. pIX clones are shown in blue and pIII clones in purple. (**b**) The table summarizes the IGHV gene segment usage, expression yield and Tm of the scFv clones. The expression yield of each clone is given as mg obtained after protein L purification of 1 L culture. Tm of the clones was determined by DSF (n = 2–4). ScFvs expressed and purified separately were run in duplicates and the results are given as mean ± SD.

Next, we expressed and purified the highest and lowest ranking scFv clones from pIII LV (57AE07 and 57AE06, respectively) and pIX HV (57AE16 and 57AE20, respectively) based on the OMV binding strength measured with crude periplasmic extracts (Supplementary Fig. 6). Both scFv clones from pIX HV and one of the clones from pIII LV used IGHV3, which was overrepresented among the clones selected from the pIX HV library. The other pIII LV clones used IGHV4, which was overrepresented in pIII LV. We then repeated the OMV binding analysis. In line with the results reported above (Fig. 7d), both pIX clones bound stronger to OMV than the two pIII clones (Fig. 8a and Supplementary Fig. 6).

As an indirect measurement of protein stability, we monitored the yield of each clone after protein L purification. The three scFvs using the generally more stable IGHV3 were expressed with much greater yield than the scFv using IGHV4 (Fig. 8b)^26^. In particular, the pIII LV-derived clone 57AE06 was expressed at an exceptionally high level, but bound only weakly to OMV (Fig. 8a).

Finally, we performed differential scanning fluorimetry (DSF) to determine thermal stability^27^. The best OMV-binding clone, 57AE20, also had the highest melting temperature (Tm) (64.5 °C), followed by the pIII LV clone, 57AE07 (62.4 °C) (Fig. 8b and Supplementary Fig. 6). 57AE16 had the lowest Tm (56.8 °C), in line with the finding that this clone was the only one of these four clones that lost the target binding ability upon the 55 °C thermal challenge of periplasmic samples (Fig. 7c and Supplementary Table S1).

In summary, also after purification the two pIX clones showed strong antigen binding compared to the pIII clones. Importantly, the strong antigen binding was accompanied with good expression yields, and the top candidate clone also had the highest resistance to thermal challenge.

## Discussion

In this study, we demonstrate that the choice of capsid protein for antibody phage display has a profound impact on the selection of specific and stable binders of good affinity. Previous studies have mainly focused on improving the antibody scaffold itself for the same purpose^3^. Here, we demonstrate superior performance of pIX display in two independent library selections employing unmodified, endogenously derived antibody V gene repertoires from mouse and humans, and find that the performance of pIX strongly benefits from HV display.

In LV display, the majority of the capsid proteins, and hence the virions, are devoid of a fusion altogether^28^. Thus, in order to retrieve a high number of binders from pIII display libraries, HV display has been shown to be beneficial. Usually, such binders exhibit reduced affinity due to avidity effects amplifying weak, yet specific interactions during selection^25^. However, the improved selection efficiency by pIX HV display did not result in low affinity clones, and we even selected clones with higher affinity from pIX HV than from pIII LV. This may be related to the physical properties of the pIX capsid as found embedded into the virion body^29^. Full-length pIII, as used here, is a large multi-domain protein shown to protrude from the virion^30^, allowing considerable rotational and positional flexibility of a fusion^31^. The rigid and confined topology of the small 3.7 kDa pIX may give less opportunity for simultaneous engagement of multiple ligand molecules by each virion in HV antibody display^4^. Thus, avidity effects may be less pronounced for pIX HV display than for pIII HV display.

Noteworthy, the pIII HV library contained a very high proportion of polyphages. This finding in combination with avidity effects likely explains the high reactivity of pIII HV in the OMV phage ELISA, where even very low affinity clones gave a detectable signal due to amplification caused by the greatly increased number of pVIII molecules available to the detection antibody. By contrast, the binders selected by the pIX HV route had high target reactivity independently of whether the scFvs were displayed on phage, or expressed as soluble molecules. This was particularly evident in the soluble scFv screen where the target reactivity of the pIX HV-derived clones greatly exceeded the reactivity of those selected from the pIII LV route. Importantly, this effect observed in screening of crude periplasmic extracts was also mirrored in analyses using purified monomeric scFvs, where the pIX HV-derived clones bound stronger to OMV than the pIII LV-derived clones.

We observed capsid-dependent effects on host cell growth during protein expression and phagemid packaging. The exact cause of this remains unclear. However, the negative impact of the SEC-directing signal sequence pelB was evident. Both pIII and pIX with signal sequence suffered from growth interference, while pIX without a signal sequence did not. The growth interference translated into enrichment of non-functional clones during library amplification. There are conflicting reports regarding the ability of pIII HV display to positively select for ORFs^32^,^33^, and we did not observe such an effect for pIII HV in the mAb 679-14-E06 selection, whereas it was pronounced for pIX HV.

Signal sequence-mediated periplasmic targeting has been shown to affect display of non-antibody pIII fusions^34^,^35^. Here, the beneficial effects of pIX display was revealed only when pIX was utilized without the pelB signal sequence. Thus, the consequence of adding a pelB sequence could well be a re-routing from the YidC, the suggested route for native pIX^13^, to the SEC translocon. Moreover, the favorable properties appear to be unique to pIX, as M13 pVIII that also depends on YidC has been shown to be inferior to pIII in antibody affinity selection^36^,^37^.

The importance of manufacturability has resulted in a strong focus on selection of stable antibody lead candidates^3^,^38^. Although the end-use format for most scFvs is full-length antibodies, transfer of the intrinsic properties from the scFv to the full-length molecule^39^,^40^ underscores the importance of selecting stable scFv candidates which limits the need for post-selection engineering^41^,^42^,^43^,^44^. Interestingly, pIX HV-selected clones predominantly utilized the more stable IGHV3 gene family, while the less stable gene family IGHV4 occurred frequently in clones selected by pIII LV^26^. In part, this may account for the better thermodynamic stability of the pIX HV clones, translating into a higher retained antigen binding after heat challenge compared to the clones selected through pIII LV. However, large variations may exist resulting in clonal deviation from such germline intrinsic properties due to distinct somatic contribution both in framework and CDR3 loops^45^. This may in part account for the lack of good binders in the IGHV3-dominated pIII HV pool. In contrast to the known distinct intrinsic differences in the thermodynamic stability of the various IGHV segments, the properties of IGKV segments are more similar, and thus contribute less to scFv stability^26^.

When we expressed and purified monomeric scFvs, we observed that the three clones using IGHV3 were produced in higher yields than the clone using IGHV4. The two pIX-derived clones (both using IGHV3) combined favorable expression characteristics with high antigen reactivity. In contrast, the pIII-derived clone, 57AE06 (using IGHV3) was expressed extremely well, but bound poorly to the antigen, indicating selection largely based on favorable expression characteristics. Although expression and stability are closely connected^46^,^47^, it did not appear to be the case with this clone. This was even more pronounced for the other pIII clone, which had a high thermal stability, but was expressed at very low level. Preferential amplification bias may obscure retrieval of desirable clones during library screening and has been reported for pIII libraries^6^,^48^. Our data indicate that the pIX-derived clones were selected and enriched primarily based on favorable target interaction, and therefore appear to be less affected by such target-unrelated effects.

In conclusion, we show that the use of signal sequence-independent pIX display translates into superior performance in antibody affinity selection. Stronger enrichment of specific clones, as well as selection of clones of higher stability and affinity was observed with pIX compared with pIII display. These benefits could in part be accounted for by less host toxicity and in part be attributed to the observation that V genes with known favorable biophysical properties were preferentially selected in a display capsid-dependent manner, even though the libraries were constructed from the same V gene repertoire. However, the most striking observation was the strong dependence on HV display. This is the first report of multivalent antibody pIX display in *de novo* library selection, explaining why the superior properties of pIX have not been revealed previously^16^.

## Methods

### Phage library generation

#### Library 1

An immune murine scFv phage library was constructed in the phagemids pFKPDN^49^ and pGALD9ΔLFN^18^, enabling display on pIII and pIX, respectively, along with co-expression of the periplasmic chaperone FkpA. Two TG2 knockout mice^50^ were immunized intraperitoneally (i.p.) with 200 μg of 679-14-E06 Fab specific for human TG2 in complete Freund’s adjuvant on day 0 followed by two booster immunizations of 100 μg Fab fragment in incomplete Freund’s adjuvant i.p. on day 18 and day 39. After receiving a final i.p. injection of 50 μg Fab fragment in PBS on day 60, serum was collected on day 63 and tested for reactivity towards the mAb in ELISA. In brief, microtiter plates were coated with 0.3 μg 679-14-E06 Fab in 50 μl PBS, washed with PBS with 0.05% (v/v) Tween 20 (PBST) and incubated with serum samples diluted as indicated in PBST. Serum from a non-immunized TG2 knockout mouse was used as a control. Detection of bound serum antibodies was done with AP-conjugated goat anti-mouse IgG (Abcam). After addition of phosphatase substrate, absorbance was measured at 405 nm in a microplate reader.

Spleens were harvested, and B cells were purified using a mouse B cell isolation kit (Miltenyi). Total RNA was isolated using RNeasy minikit (QIAGEN) and cDNA was synthesized using random hexamer primers (Invitrogen). V genes were amplified using a modified Krebber protocol where assembly of scFvs was done sequentially, first by ligation and transformation of the V_L_ followed by ligation and transformation of V_H_^51^. Briefly, the primers were redesigned to accommodate the pGALD9ΔLFN/pFKPDN phagemids by keeping the V gene-specific region only for a primary amplification, followed by a secondary amplification adding new RE-sites (NcoI/HindIII for V_H_ and MluI/NotI for V_L_). Correct bands from the primary PCR were extracted and purified from an agarose gel, followed by re-amplification using the secondary primer set to add RE-sites. Extracted bands were digested and purified before the V_L_ fragments were ligated into phosphatase treated vectors at 16 °C overnight. 40 ng of library DNA was transformed into *E. coli* AVB100mkII^52^ using cell aliquots of 350 μl and an ECM 600 electroporator (BTX) as described^53^. The transformations were plated on Bio-Assay dishes (Nunc) and incubated overnight at 30 °C. Colonies were scraped from the plates and phagemid DNA isolated. The procedure was repeated for the V_H_ fragments. Both the pIII and pIX libraries reached a diversity of ~3 × 10^6^ (determined by transformation output). Phagemid rescue was done by inoculating scraped material to OD600 nm 0.05 in 2x YT supplemented with 30 μg/ml tetracycline, 100 μg/ml ampicillin and 0.1 M glucose (2x YT-TAG) and incubated with rigorous shaking at 37 °C until OD reached 0.1–0.2. The cultures were superinfected with helperphage at MOI20 and incubated with gentle shaking at 37 °C for 60 min, followed by rigorous shaking for 30 min, before centrifugation and medium replacement to 2x YT supplemented with 100 μg/ml ampicillin and 50 μg/ml kanamycin (2x YT-AK) and incubation further 7 h at 30 °C. Phage particles were purified and concentrated by 2x PEG/NaCl precipitation and resuspended in PBS and cfu was determined by spot titration^54^. Both libraries were rescued with M13K07 (GE Healthcare) for LV display. In addition, the pIII-library was rescued with Hyperphage^21^ (Progen Biotechnik), and the pIX library was rescued with DeltaPhage^19^, resulting in pIII and pIX HV display libraries, respectively. Animal experiment procedures were approved by the National Committee for Animal Experiments (Oslo, Norway) and the methods were carried out in accordance with these approved guidelines and regulations.

#### Library 2

A naïve human scFv-pIII phage library (pL-NBLκ)^22^ was reformatted to the phagemids used for library 1. The original library was assembled from the IgD and IgM repertoire of 6 healthy individuals and pre-selected on protein L to enrich functional scFv clones (final diversity of approx. 3 × 10^8^). The scFv cassette was PCR amplified directly from the phage library (1 μl PEG precipitated phage stock containing ~6.6 × 10^9^ virions) using 0.25 μM each of forward 5′-CTCAGCCGGCCATGGCC-3′ and reverse 5′-TTTGGATCCAGCGGCCGC-3′ biotinylated primers (MWG Operon) containing NcoI and NotI RE-sites and 0.02 U Herculase II Fusion DNA polymerase. The correct band was extracted from an agarose gel, followed by digestion and capture of biotinylated ends using MyOne Streptavidin T1 magnetic beads (Invitrogen). scFv cassettes were purified and ligated into phosphatase-treated vectors in the presence of polynucleotide kinase at 16 °C overnight, followed by purification using Pellet Paint Coprecipitant (Novagen). 80–400 ng library DNA was transformed into *E. coli* SS320 (Lucigen) as described for library 1. pIII and pIX libraries (pIII-rpL-NBLκ and pIX-rpL-NBLκ) obtained 1.95 × 10^9^ and 1.85 × 10^9^ primary transformants, respectively. Phage rescue, PEG/NaCl precipitation and spot titration was performed as described for Library 1.

### Phage display selection and recovery of binders

Selections were performed using immunotubes (NUNC) coated with antigen overnight at 4 °C, either 0.1 μg/ml mAb 679-14-E06, 5 μg/ml of each of the negative selection mAbs 679-14-A05 (share IGHV with mAb 679-14-E06) and 693-1-A03 (share IGKV with mAb 679-14-E06), or OMV (of *N. meningitidis* group B 44/76)^55^ in 4 ml PBS. In the OMV selection, antigen concentration was decreased 10-fold in each round, starting at 10 μg/ml in R1, and washing stringency increased from 5x PBST + 5x PBS in R1, 10x + 10x in R2 and 20x + 20x in R3. In the selection against mAb 679-14-E06, the phage libraries were first incubated in tubes coated with the two negative selection mAbs, before the supernatant containing unbound library members were transferred to the mAb 679-14-E06 coated tubes. Antigen concentration and washing (10x PBST + 10x PBS) was kept constant. In both selections, the phage libraries were incubated with antigen for 1 h at room temperature using a rotating device. Tubes were briefly vortexed between each wash. 4% (w/v) non-fat skim milk powder (PBSM) or 2% (w/v) bovine serum albumin (essentially fatty acid free) was used as blocking reagents in alternating selection rounds. Elution was performed by 10 min incubation with 0.5 ml 0.5% trypsin followed by *E. coli* infection using half of the eluate. A small sample of the infected culture was removed for determination of output.

### Reformatting and expression of single library clones

#### Single-clone phage expression

Briefly, single clones were inoculated into 400 μl 2x YT-TAG in 96-deep well plates and incubated overnight in a Titramax (Heidolph). 10 μl were transferred to new 2x YT-TAG-containing plates and incubated for 3 h at 37 °C before addition of either Hyperphage or Deltaphage (10^9^ cfu/well), followed by gentle shaking at 37 °C for 30 min, and 30 min with rigorous shaking before medium replacement to 2x YT-AK and overnight incubation at 30 °C. Phage supernatants harvested after centrifugation were used directly in ELISA.

#### Single-clone soluble expression

The scFv cassette was batch-cloned as NcoI/NotI fragments from the phagemids into pFKPEN^56^, placing the scFvs in-frame with c-myc and his-tags, and transformed into *E. coli* XL1-Blue (Stratagene). Individual clones were inoculated and expressed in 96-deep well plates using 400 μl culture medium for screening experiments or in either 10 ml or 200 ml cultures for larger-scale expressions. These were performed essentially as described, except that 2x YT was used as culture medium and 0.1 mM IPTG was used for induction of protein expression in small scale expressions^56^. For 96-deep well expression 50 μl was used to re-inoculated new cultures after overnight growth. Single clones identified by screening were sequenced by GATC Biotech.

For purification of OMV-reactive scFvs, periplasmic samples were diluted 4x with PBS and pH adjusted to 7.4, before affinity purification using a HiTrap Protein L column (GE Healthcare). Proteins were eluted with 0.1 M Glycine-HCl pH 2.7 and neutralized by addition of 1 M Tris-HCl pH 8. For size exclusion chromatography, Superdex 200 Increase 10/300 GL run in PBS supplemented with 150 mM NaCl was used. pH was adjusted according to the pI of the individual samples.

### ELISA

Microtiter plates were coated overnight at 4 °C with anti-TG2 mAbs (1 μg/ml), OMV (5 μg/ml) or BSA-phOx (1 μg/ml) in PBS before blocking with PBSM. OMV was coated at 0.4 μg/ml in ELISAs with purified OMV-reactive scFvs. Phage, equal amounts of combined medium and periplasmic fractions containing scFvs, or 0.5 μg/ml hIgG1 diluted in PBST were added and detected with either anti-M13-HRP (Amersham Biosciences, 1:5000), anti-His-tag-HRP (AbD Serotech, 1:5000), anti-c-myc tag (Invitrogen, 1:5000) or polyclonal anti-human IgG Fc-AP (Sigma, 1:2000) in PBST, respectively. In the case of anti-c-myc detection, anti-mouse IgG-HRP (Dako, 1:2000) was used as secondary antibody. HRP ELISAs were developed by addition of TMB solution, while AP ELISAs were developed with 1 mg/ml phosphatase substrate in diethanolamine buffer before absorbance reading at 620 nm (450 nm in the case of HCl addition) or 405 nm, respectively. Assays were performed at room temperature in duplicates, except for single clone screenings with only one well/sample. Between each layer, the plates were washed 3x with PBST. For temperature-challenge assays, medium and periplasmic fractions were heat-challenged for 10 min at 55 °C before they were cooled and analyzed by ELISA. In some cases, medium and periplasm samples were directly coated before blocking. Mouse anti-c-myc tag followed by HRP-conjugated anti-mouse IgG was used to detect the c-myc tag, while HRP-conjugated protein L (Genscript, 1:2000) was used to detect conformationally intact folded scFv.

### Monitoring of *E. coli* growth characteristics

To monitor *E. coli* growth curve, overnight cultures were inoculated to OD600 nm 0.025 in 2x YT-TAG and grown overnight at 37 °C. Alternatively, after 1.5 h incubation at 37 °C, the cultures were super-infected by helper phage and phage packaged as described above. OD600 nm was measured at regular intervals.

### SDS-PAGE and Western blot

10 μl medium, periplasmic and cytosolic samples were heated at 95 °C for 5 min with BOLT^TM^ LDS sample buffer before separation on 12% NuPAGE BT gels in Bolt MES SDS running buffer (reagents from Novex) at 165 V for 35 min along with Broad-range ladder. Proteins were either coomassie stained or blotted onto PVDF membrane in Tris-Glycine buffer (25 mM Tris, 192 mM Glycine, 20% methanol, pH 8.3) using a semi-dry blotting apparatus. Membranes were blocked with PBSM before detection with mouse anti-c-myc tag followed by anti-mouse IgG-HRP diluted in PBSTM. Membranes were developed using TMB Insoluble. For phage Western blots, normalized phage samples of 2 × 10^9^ cfu^AmpR^/lane and 1 × 10^10^ cfu^AmpR^/lane was used for pIII and pIX blots, respectively. Mouse anti-pIII (MoBiTec, 1:5000) and anti-mouse IgG-HRP (1:10.000) was used for pIII blots. A polyclonal anti-pIX rabbit serum was generated by immunization using a peptide (*N*-CITYFTRLMETSS-*C*) of the C-terminal pIX portion (AbMART). The validated anti-pIX serum was used at 1:2000 in combination with anti-rabbit IgG-HRP (1:5000) for pIX blots. Western blots were detected by reading chemiluminescent signals.

### Reformatting to IgG and eukaryotic protein expression and purification

Synthetic gene fragments encoding V_H_ and V_L_ of the selected mAb 2G9 together with intronic splice donor sites (Genscript) were cloned as BsmI-BsiWi fragments into the IgG genomic expression vectors pLNOH2_NIP_ and pLNOk_NIP_ 57, encoding constant human gamma1 with N297G mutation and constant human kappa domains, respectively, exchanging the existing specificity for the hapten NIP. hIgG1 mAb 2G9 was expressed as previously described^58^ and purified using CaptureSelect anti-human CH1 (GE Healthcare). Before SPR, a fraction was further purified by size exclusion chromatography using Superdex 200 (GE Healthcare). Anti-TG2 antibodies and Fab fragments were produced in HEK293F cells (ATCC) as previously described^20^,^59^ and subsequently purified from supernatants using Protein G Sepharose or Protein L Sepharose (GE Healthcare), respectively.

### Flow cytometry of transduced cell lines

A20 lymphoma cells expressing the TG2 reactive 679-14-E06 BCR or control BCR not reactive to TG2 (693-2-F02) were generated and maintained as previously described^60^. For flow cytometric analysis, cells were pre-blocked with mouse Seroblock (Serotec) and stained with hIgG1 anti-679-14-E06 mAb 2G9 (2 μg/ml in PBS + 2% FCS) followed by anti-human IgG1-PE (1 μg/ml; Acris GmbH) for 30 min on ice. Cells were analyzed on a FACSCalibur instrument (BD Biosciences). The hIgG1 mAb 2G9 and isotype control hIgG1 were titrated at the indicated concentrations. EC_50_ was determined using the “dose-response (stimulation)”-equation in GraphPad Prism 5.

### Transmission electron microscopy

2x PEG precipitated virion samples normalized to 1 × 10^10^ virions/ml were prepared for microscopy as described^18^. Images were recorded in a Philips CM100 transmission electron microscope at 80 kV using a Olympus Quemesa camera or in a JEOL1400Plus at 80 kV using a Ruby camera. A measuring grid was overlayed and intersections with the grid were counted. The length of each particle was calculated using π/4 * I * d (I being the number of intersections and d being the distance between the grid lines)^61^.

### Surface plasmon resonance measurement and analysis

mAb 2G9 was diluted in acetate buffer pH 4.5 and immobilized on a CM3 Series S sensor chip by amine coupling to 550 resonance units (RU) using a Biacore T200 (GE Healthcare). A control antibody was immobilized on the reference flow cell. Binding kinetics were measured using single-cycle kinetics with a 3-fold dilution from 1 μM to 0.0123 μM Fab 679-14-E06 at a flow rate of 30 μl/min in PBS supplemented with 0.05% surfactant P20 at 25 °C. All data were reference flow cell-subtracted and evaluated with T200 evaluation software. A 1:1 Langmuir binding model was used for determination of K_D_.

### Differential scanning fluorimetry

Differential scanning fluorimetry was performed essentially as described^27^. Briefly, scFv samples were diluted to 0.1 mg/ml in 25 μl and mixed with SYPRO Orange at 1:1000. Samples were run in duplicates in a 96 well Lightcycler multiwell plate using a Lightcycler 480 Instrument II (Roche). After a stabilization period of 10 min at 25 °C, temperatures between 25 °C and 95 °C were scanned at a rate of 2 °C/min. Data was collected every 0.5 °C using the 440 nm excitation and 610 nm emission filters. Data transformation and analysis was performed as described.

## Additional Information

**How to cite this article**: Høydahl, L. S. *et al*. Multivalent pIX phage display selects for distinct and improved antibody properties. *Sci. Rep.* **6**, 39066; doi: 10.1038/srep39066 (2016).

**Publisher's note:** Springer Nature remains neutral with regard to jurisdictional claims in published maps and institutional affiliations.

## Supplementary Material

Supplementary Material

## References

[b1] Smith G. P. Filamentous fusion phage: novel expression vectors that display cloned antigens on the virion surface. Science. 228, 1315–1317 (1985). 4001944 10.1126/science.4001944

[b2] Bradbury A. R., Sidhu S., Dubel S. & McCafferty J. Beyond natural antibodies: the power of *in vitro* display technologies. Nat Biotechnol. 29, 245–254 (2011). 21390033 10.1038/nbt.1791 PMC3057417

[b3] Ponsel D., Neugebauer J., Ladetzki-Baehs K. & Tissot K. High affinity, developability and functional size: the holy grail of combinatorial antibody library generation. Molecules. 16, 3675–3700 (2011). 21540796 10.3390/molecules16053675 PMC6263270

[b4] Rakonjac J., Bennett N. J., Spagnuolo J., Gagic D. & Russel M. Filamentous Bacteriophage: Biology, Phage Display and Nanotechnology Applications. Curr Issues Mol Biol. 13, 51–76 (2011). 21502666

[b5] Løset G. Å. & Sandlie I. Next generation phage display by use of pVII and pIX as display scaffolds. Methods. 58, 40–46 (2012). 22819858 10.1016/j.ymeth.2012.07.005

[b6] de Bruin R., Spelt K., Mol J., Koes R. & Quattrocchio F. Selection of high-affinity phage antibodies from phage display libraries. Nat Biotechnol. 17, 397–399 (1999). 10207892 10.1038/7959

[b7] Derda R. . Diversity of phage-displayed libraries of peptides during panning and amplification. Molecules. 16, 1776–1803 (2011). 21339712 10.3390/molecules16021776 PMC6259649

[b8] Matochko W. L. . Deep sequencing analysis of phage libraries using Illumina platform. Methods. 58, 47–55 (2012). 22819855 10.1016/j.ymeth.2012.07.006

[b9] Gao C. . A method for the generation of combinatorial antibody libraries using pIX phage display. Proc Natl Acad Sci USA 99, 12612–12616 (2002). 12239343 10.1073/pnas.192467999 PMC130508

[b10] Tornetta M. . Antibody Fab display and selection through fusion to the pIX coat protein of filamentous phage. J Immunol Methods. 360, 39–46 (2010). 20600082 10.1016/j.jim.2010.06.001

[b11] Shi L. . De novo selection of high-affinity antibodies from synthetic fab libraries displayed on phage as pIX fusion proteins. J Mol Biol. 397, 385–396 (2010). 20114051 10.1016/j.jmb.2010.01.034

[b12] Simons G. F., Konings R. N. & Schoenmakers J. G. Genes VI, VII, and IX of phage M13 code for minor capsid proteins of the virion. Proc Natl Acad Sci USA 78, 4194–4198 (1981). 6945579 10.1073/pnas.78.7.4194 PMC319755

[b13] Ploss M. & Kuhn A. Membrane insertion and assembly of epitope-tagged gp9 at the tip of the M13 phage. BMC Microbiol. 11, 211, 10.1186/1471-2180-11-211 (2011). 21943062 PMC3193035

[b14] Endemann H. & Model P. Location of filamentous phage minor coat proteins in phage and in infected cells. J Mol Biol. 250, 496–506 (1995). 7616570 10.1006/jmbi.1995.0393

[b15] Gao C. . Making artificial antibodies: a format for phage display of combinatorial heterodimeric arrays. Proc Natl Acad Sci USA 96, 6025–6030 (1999). 10339535 10.1073/pnas.96.11.6025 PMC26829

[b16] Huovinen T. . The selection performance of an antibody library displayed on filamentous phage coat proteins p9, p3 and truncated p3. BMC Res Notes. 7, 661, 10.1186/1756-0500-7-661 (2014). 25238965 PMC4176855

[b17] Tornetta M., Reddy R. & Wheeler J. C. Selection and maturation of antibodies by phage display through fusion to pIX. Methods. 58, 34–39 (2012). 22841960 10.1016/j.ymeth.2012.07.010

[b18] Løset G. Å., Roos N., Bogen B. & Sandlie I. Expanding the versatility of phage display II: improved affinity selection of folded domains on protein VII and IX of the filamentous phage. PLoS One. 6, e17433, 10.1371/journal.pone.0017433 (2011). 21390283 PMC3044770

[b19] Nilssen N. R. . DeltaPhage–a novel helper phage for high-valence pIX phagemid display. Nucleic Acids Res. 40, e120, 10.1093/nar/gks341 (2012). 22539265 PMC3439877

[b20] Di Niro R. . High abundance of plasma cells secreting transglutaminase 2-specific IgA autoantibodies with limited somatic hypermutation in celiac disease intestinal lesions. Nat Med. 18, 441–445 (2012). 22366952 10.1038/nm.2656 PMC4533878

[b21] Rondot S., Koch J., Breitling F. & Dubel S. A helper phage to improve single-chain antibody presentation in phage display. Nat Biotechnol. 19, 75–78 (2001). 11135557 10.1038/83567

[b22] Løset G. Å. . Construction, evaluation and refinement of a large human antibody phage library based on the IgD and IgM variable gene repertoire. J Immunol Methods. 299, 47–62 (2005). 15914190 10.1016/j.jim.2005.01.014

[b23] Hayhurst A. . Isolation and expression of recombinant antibody fragments to the biological warfare pathogen Brucella melitensis. J Immunol Methods. 276, 185–196 (2003). 12738372 10.1016/s0022-1759(03)00100-5

[b24] Lillo A. M. . Development of phage-based single chain Fv antibody reagents for detection of Yersinia pestis. PLoS One. 6, e27756, 10.1371/journal.pone.0027756 (2011). 22174746 PMC3234238

[b25] O’Connell D., Becerril B., Roy-Burman A., Daws M. & Marks J. D. Phage versus phagemid libraries for generation of human monoclonal antibodies. J Mol Biol. 321, 49–56 (2002). 12139932 10.1016/s0022-2836(02)00561-2

[b26] Ewert S., Huber T., Honegger A. & Pluckthun A. Biophysical properties of human antibody variable domains. J Mol Biol. 325, 531–553 (2003). 12498801 10.1016/s0022-2836(02)01237-8

[b27] Niesen F. H., Berglund H. & Vedadi M. The use of differential scanning fluorimetry to detect ligand interactions that promote protein stability. Nat Protoc. 2, 2212–2221 (2007). 17853878 10.1038/nprot.2007.321

[b28] Bradbury A. R. & Marks J. D. Antibodies from phage antibody libraries. J Immunol Methods. 290, 29–49 (2004). 15261570 10.1016/j.jim.2004.04.007

[b29] Makowski L. Structural constraints on the display of foreign peptides on filamentous bacteriophages. Gene. 128, 5–11 (1993). 8508959 10.1016/0378-1119(93)90146-t

[b30] Gray C. W., Brown R. S. & Marvin D. A. Adsorption complex of filamentous fd virus. J Mol Biol. 146, 621–627 (1981). 7024557 10.1016/0022-2836(81)90050-4

[b31] Holliger P., Riechmann L. & Williams R. L. Crystal structure of the two N-terminal domains of g3p from filamentous phage fd at 1.9 A: evidence for conformational lability. J Mol Biol. 288, 649–657 (1999). 10329170 10.1006/jmbi.1999.2720

[b32] Brockmann E. C. . Synthetic single-framework antibody library integrated with rapid affinity maturation by VL shuffling. Protein Eng Des Sel. 24, 691–700 (2011). 21680620 10.1093/protein/gzr023

[b33] Hust M. . Enrichment of open reading frames presented on bacteriophage M13 using hyperphage. Biotechniques. 41, 335–342 (2006). 16989094 10.2144/000112225

[b34] Steiner D., Forrer P., Stumpp M. T. & Pluckthun A. Signal sequences directing cotranslational translocation expand the range of proteins amenable to phage display. Nat Biotechnol. 24, 823–831 (2006). 16823375 10.1038/nbt1218

[b35] Thie H., Schirrmann T., Paschke M., Dubel S. & Hust M. SRP and Sec pathway leader peptides for antibody phage display and antibody fragment production in *E. coli*. N Biotechnol. 25, 49–54 (2008). 18504019 10.1016/j.nbt.2008.01.001

[b36] Kretzschmar T. & Geiser M. Evaluation of antibodies fused to minor coat protein III and major coat protein VIII of bacteriophage M13. Gene. 155, 61–65 (1995). 7698668 10.1016/0378-1119(94)00897-2

[b37] Samuelson J. C. . YidC mediates membrane protein insertion in bacteria. Nature. 406, 637–641 (2000). 10949305 10.1038/35020586

[b38] Dudgeon K., Rouet R., Famm K. & Christ D. Selection of human VH single domains with improved biophysical properties by phage display. Methods Mol Biol. 911, 383–397 (2012). 22886264 10.1007/978-1-61779-968-6_23

[b39] Schaefer J. V. & Pluckthun A. Transfer of engineered biophysical properties between different antibody formats and expression systems. Protein Eng Des Sel. 25, 485–506 (2012). 22763265 10.1093/protein/gzs039

[b40] Rothlisberger D., Honegger A. & Pluckthun A. Domain interactions in the Fab fragment: a comparative evaluation of the single-chain Fv and Fab format engineered with variable domains of different stability. J Mol Biol. 347, 773–789 (2005). 15769469 10.1016/j.jmb.2005.01.053

[b41] Rouet R., Lowe D. & Christ D. Stability engineering of the human antibody repertoire. FEBS Lett. 588, 269–277 (2014). 24291820 10.1016/j.febslet.2013.11.029

[b42] Shealy D. J. . Characterization of golimumab, a human monoclonal antibody specific for human tumor necrosis factor alpha. MAbs. 2, 428–439 (2010). 20519961 10.4161/mabs.2.4.12304 PMC3180089

[b43] Kehoe J. W. . Isolation and optimization for affinity and biophysical characteristics of anti-CCL17 antibodies from the VH1-69 germline gene. Protein Eng Des Sel. 27, 199–206 (2014). 24742503 10.1093/protein/gzu012

[b44] Entzminger K. C., Johnson J. L., Hyun J., Lieberman R. L. & Maynard J. A. Increased Fab thermoresistance via VH-targeted directed evolution. Protein Eng Des Sel. 28, 365–377 (2015). 26283664 10.1093/protein/gzv037 PMC4596279

[b45] Jespers L., Schon O., Famm K. & Winter G. Aggregation-resistant domain antibodies selected on phage by heat denaturation. Nat Biotechnol. 22, 1161–1165 (2004). 15300256 10.1038/nbt1000

[b46] Ewert S., Honegger A. & Pluckthun A. Structure-based improvement of the biophysical properties of immunoglobulin VH domains with a generalizable approach. Biochemistry. 42, 1517–1528 (2003). 12578364 10.1021/bi026448p

[b47] Gunnarsen K. S. . Chaperone-assisted thermostability engineering of a soluble T cell receptor using phage display. Sci Rep. 3, 1162, 10.1038/srep01162 (2013). 23362461 PMC3557450

[b48] Matochko W. L., Cory Li, S., Tang S. K. & Derda R. Prospective identification of parasitic sequences in phage display screens. Nucleic Acids Res. 42, 1784–1798 (2014). 24217917 10.1093/nar/gkt1104 PMC3919620

[b49] Løset G. Å., Lunde E., Bogen B., Brekke O. H. & Sandlie I. Functional phage display of two murine alpha/beta T-cell receptors is strongly dependent on fusion format, mode and periplasmic folding assistance. Protein Eng Des Sel. 20, 461–472 (2007). 17925331 10.1093/protein/gzm044

[b50] De Laurenzi V. & Melino G. Gene disruption of tissue transglutaminase. Mol Cell Biol. 21, 148–155 (2001). 11113189 10.1128/MCB.21.1.148-155.2001 PMC88788

[b51] Krebber A. . Reliable cloning of functional antibody variable domains from hybridomas and spleen cell repertoires employing a reengineered phage display system. J Immunol Methods. 201, 35–55 (1997). 9032408 10.1016/s0022-1759(96)00208-6

[b52] Løset G. Å., Bogen B. & Sandlie I. Expanding the versatility of phage display I: efficient display of peptide-tags on protein VII of the filamentous phage. PLoS One. 6, e14702, 10.1371/journal.pone.0014702 (2011). 21390217 PMC3044727

[b53] Tonikian R., Zhang Y., Boone C. & Sidhu S. S. Identifying specificity profiles for peptide recognition modules from phage-displayed peptide libraries. Nat Protoc. 2, 1368–1386 (2007). 17545975 10.1038/nprot.2007.151

[b54] Løset G. Å., Kristinsson S. G. & Sandlie I. Reliable titration of filamentous bacteriophages independent of pIII fusion moiety and genome size by using trypsin to restore wild-type pIII phenotype. Biotechniques. 44, 551–552, 554 (2008). 18476820 10.2144/000112724

[b55] Fredriksen J. H. . Production, characterization and control of MenB-vaccine “Folkehelsa”: an outer membrane vesicle vaccine against group B meningococcal disease. NIPH Ann. 14, 67–79, discussion 79–80 (1991). 1812438

[b56] Gunnarsen K. S. . Periplasmic expression of soluble single chain T cell receptors is rescued by the chaperone FkpA. BMC Biotechnol. 10, 8, 10.1186/1472-6750-10-8 (2010). 20128915 PMC2834602

[b57] Norderhaug L., Olafsen T., Michaelsen T. E. & Sandlie I. Versatile vectors for transient and stable expression of recombinant antibody molecules in mammalian cells. J Immunol Methods. 204, 77–87 (1997). 9202712 10.1016/s0022-1759(97)00034-3

[b58] Berntzen G. . Prolonged and increased expression of soluble Fc receptors, IgG and a TCR-Ig fusion protein by transiently transfected adherent 293E cells. J Immunol Methods. 298, 93–104 (2005). 15847800 10.1016/j.jim.2005.01.002

[b59] Chen X. . Structural Basis for Antigen Recognition by Transglutaminase 2-specific Autoantibodies in Celiac Disease. J Biol Chem. 290, 21365–21375 (2015). 26160175 10.1074/jbc.M115.669895 PMC4571865

[b60] Stamnaes J., Iversen R., du Pre M. F., Chen X. & Sollid L. M. Enhanced B-Cell Receptor Recognition of the Autoantigen Transglutaminase 2 by Efficient Catalytic Self-Multimerization. PLoS One. 10, e0134922, 10.1371/journal.pone.0134922 (2015). 26244572 PMC4526674

[b61] Weibel E. R. Practical Methods for Biological Morphometry (Academic press, 1979).

