# Supplementary material for: Multivalent pIX phage display selects for distinct and improved antibody properties
Source: Sci Rep. 2016 Dec 14;6:39066. doi: 10.1038/srep39066 (PMC5155289; doi:10.1038/srep39066)
Supplement: Supplementary Material [file srep39066-s1.pdf]

Multivalent pIX phage display selects for distinct and improved antibody properties

Lene S. Høydahl[1,+], Nicolay R. Nilssen[1,2,+], Kristin S. Gunnarsen[1], M Fleur du Pré[1], Rasmus Iversen[1], Norbert Roos[2], Xi Chen[1], Terje E. Michaelsen[3,4], Ludvig M. Sollid[1], Inger Sandlie[1,2], and Geir Å. Løset[1,2,5,*]

[1]Centre for Immune Regulation and Department of Immunology, University of Oslo and Oslo University Hospital, N-0372 Oslo, Norway

[2]Department of Biosciences, University of Oslo, N-0316 Oslo, Norway

[3]Department of Immunology, Norwegian Institute of Public Health, N-0403 Oslo, Norway

[4]School of Pharmacy, University of Oslo, N-0316 Oslo, Norway

[5]Nextera AS, N-0349 Oslo, Norway

[+]Both authors contributed equally to this work.

*To whom correspondence should be addressed:

Tel: +47 45025421; Fax: +47 22604427; Email: g.a.loset@ibv.uio.no

Supplementary Figure S1-6 and Supplementary Table S1

Supplementary Figure S1

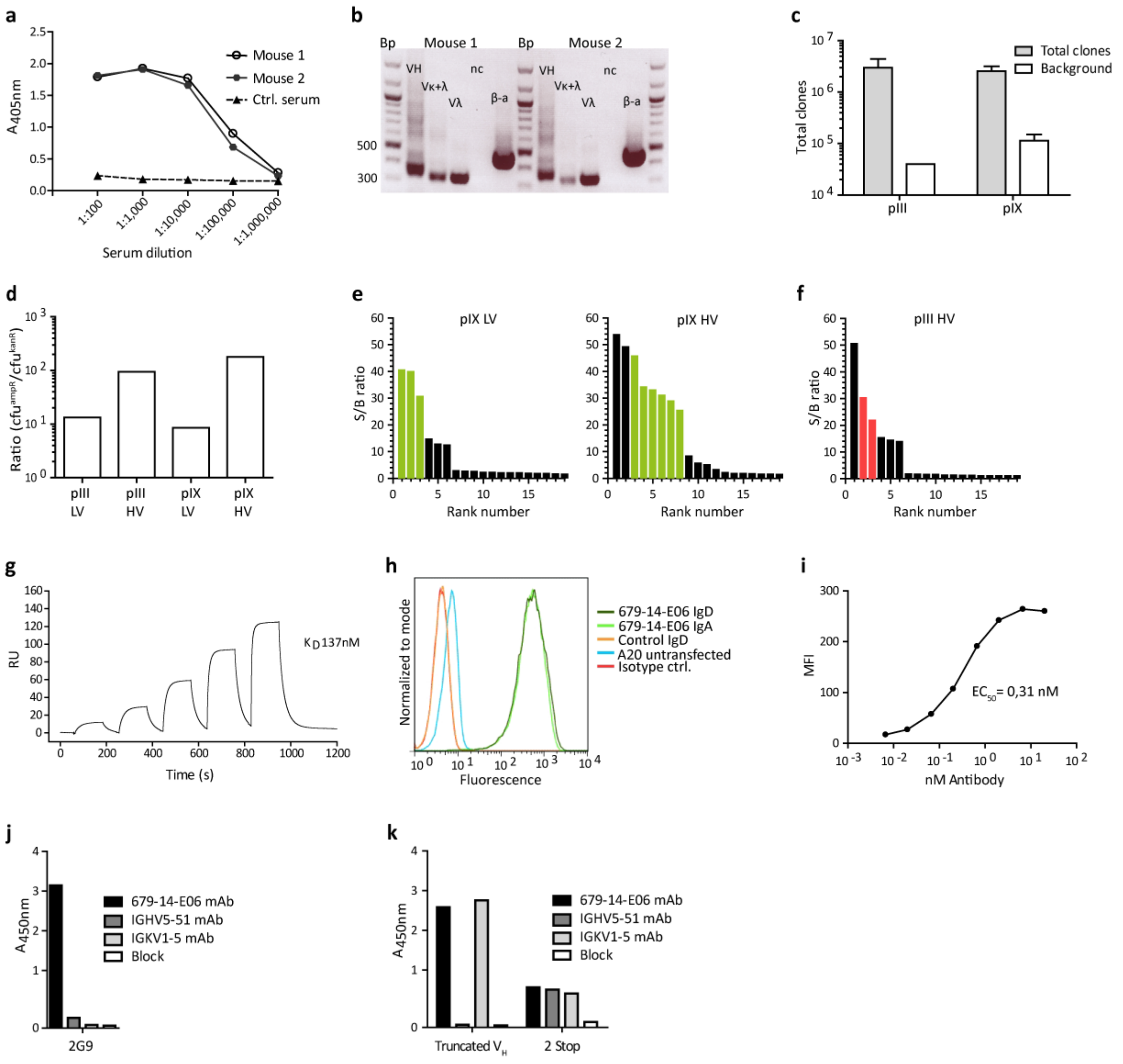

Supplementary Figure S1. Validation and characterization of the murine immune library and the selected clone 2G9. (**a**) After immunization, serum from two mice was tested for binding to the 679-14-E06 Fab fragment in ELISA. Bound serum antibodies were detected with anti-mouse IgG-ALP. (**b**) Agarose gel electrophoresis showing PCR amplification of V genes from the two mice. From left to right: 100bp ladder, V_H, V_L κ and λ mix, V_L λ, nc; negative control (no template), and β-a; mouse β-actin as a positive control. (**c**) Phage library size was determined by total number of transformants after plating a serial dilution of the library transformation mixtures in triplicates. Mean ± SD is shown.

(**d**) Phagemid/helperphage genome ratios of the R0 phage libraries determined by cfu from infectious titration. (**e**) Isolated ELISA signals from Figure 2B showing the dominant clone (green bars) in pIX libraries after two selection rounds. (**f**) Isolated signals from Figure 2B showing in-frame V_H truncations (red bars) in the pIII HV library scored as positive hits in single clone screening. (**g**) Representative SPR sensograms (n=3) showing single-cycle kinetics of 2G9 hIgG1. 2G9 was immobilized, and titrated amounts of 679-14-E06 Fab were injected. A 1:1 Langmuir binding models was used for determination of K_D. (**h**) Staining of A20 transfectants expressing 679-14-E06 IgD or IgA BCR or control IgD BCR. Cells were stained with 2G9 hIgG1 and bound IgG was detected using anti-human IgG1-PE. (**i**) Dose-response curve of 2G9 hIgG1 titration against A20 cells transfected with 679-14-E06 IgD BCR. Bound IgG was detected using anti-human IgG1-PE. The EC_{50} was determined using the “dose-response (stimulation)”-equation in the program GraphPad Prism 5. (**j-k**) The dominating clone 2G9 (**j**) or clones containing either an in-frame V_H truncation or stop codons (**k**) were analyzed for binding reactivity in ELISA against mAb 679-14-E06 or control mAbs sharing either IGHV5-51 or IGKV1-5 with mAb 679-14-E06.

Supplementary Figure S2

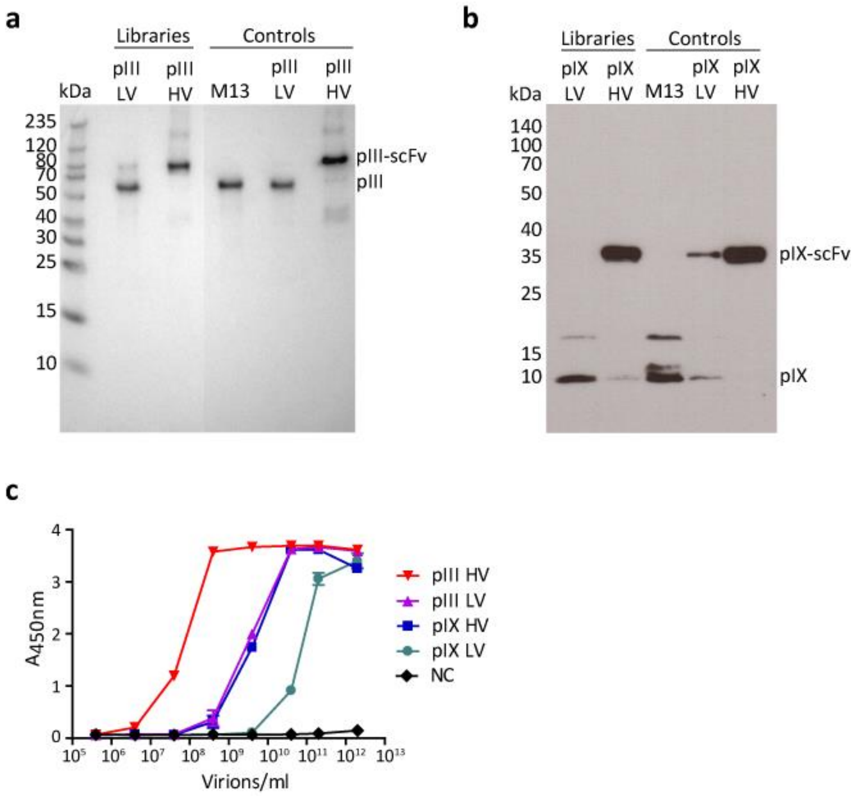

Supplementary Figure S2. HV display translates into increased antigen binding. (a and b) The different display levels of the scFv-capsid fusions achieved in LV and HV display is illustrated by western blot analysis (n=2) of normalized R0 rpL-NBLκ libraries, control phages displaying scFv anti-phOx, and M13K07 (M13). Display valence and capsid fusion protein of the R0 libraries and the control phages are indicated in the figures. Samples were detected using either (**a**) an anti-pIII antibody or (**b**) an anti-pIX antibody. (**c**) Representative ELISA showing the functional effect of increased valence. Titrated scFv anti-phOx phage samples were captured in BSA-phOx coated wells and detected using anti-M13-HRP. NC; negative control (pIX LV display of an irrelevant scFv). The results are given as mean ± SD of duplicates. A generic feature of pIX is a lower detection compared to the pIII counterpart. This is in part explained by fewer detection antibodies (anti-pVIII) binding the shorter pIX virions, and possibly reduced avidity effects of pIX-scFv fusions compared to pIII-scFv (elaborated on in the discussion).

Supplementary Figure S3

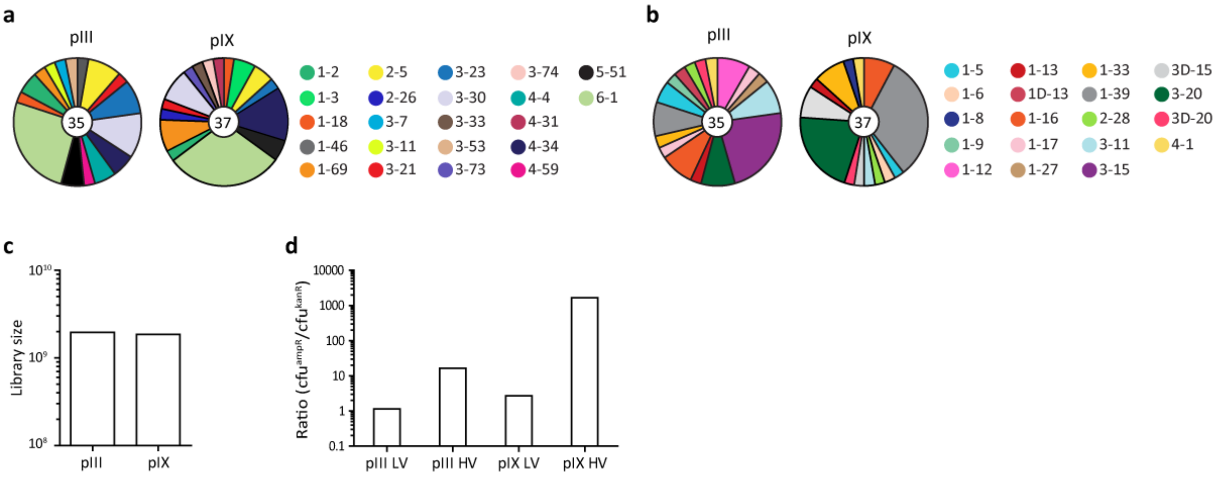

Supplementary Figure S3. Validation of the initial phage libraries and the libraries during selection. Random clones were sequenced after library transformation to evaluate library quality and V gene distribution. (**a**) IGHV usage of pIII and pIX libraries. (**b**) IGKV usage of pIII and pIX libraries. Gene segment usage was identified using the IMGT database (www.imgt.org) and only full-length functional clones were included. (**c**) Prior to reformatting the pIII and native pIX phagemids, the pL-NBL library had an anticipated diversity of about 2x 10^{8} unique clones. The graph depicts the absolute number of transformants achieved during re-formatting, which represents a 10-fold increase compared to the original library diversity, hence strongly indicating an adequate preservation of the initial clonal diversity upon retransformation. (**d**) The titer of packaged phage samples was determined by infectious titration to determine the abundance of phagemid (cfu^{ampR}) and helper phage (cfu^{kanR}) genotypes in the unselected libraries.

Supplementary Figure S4

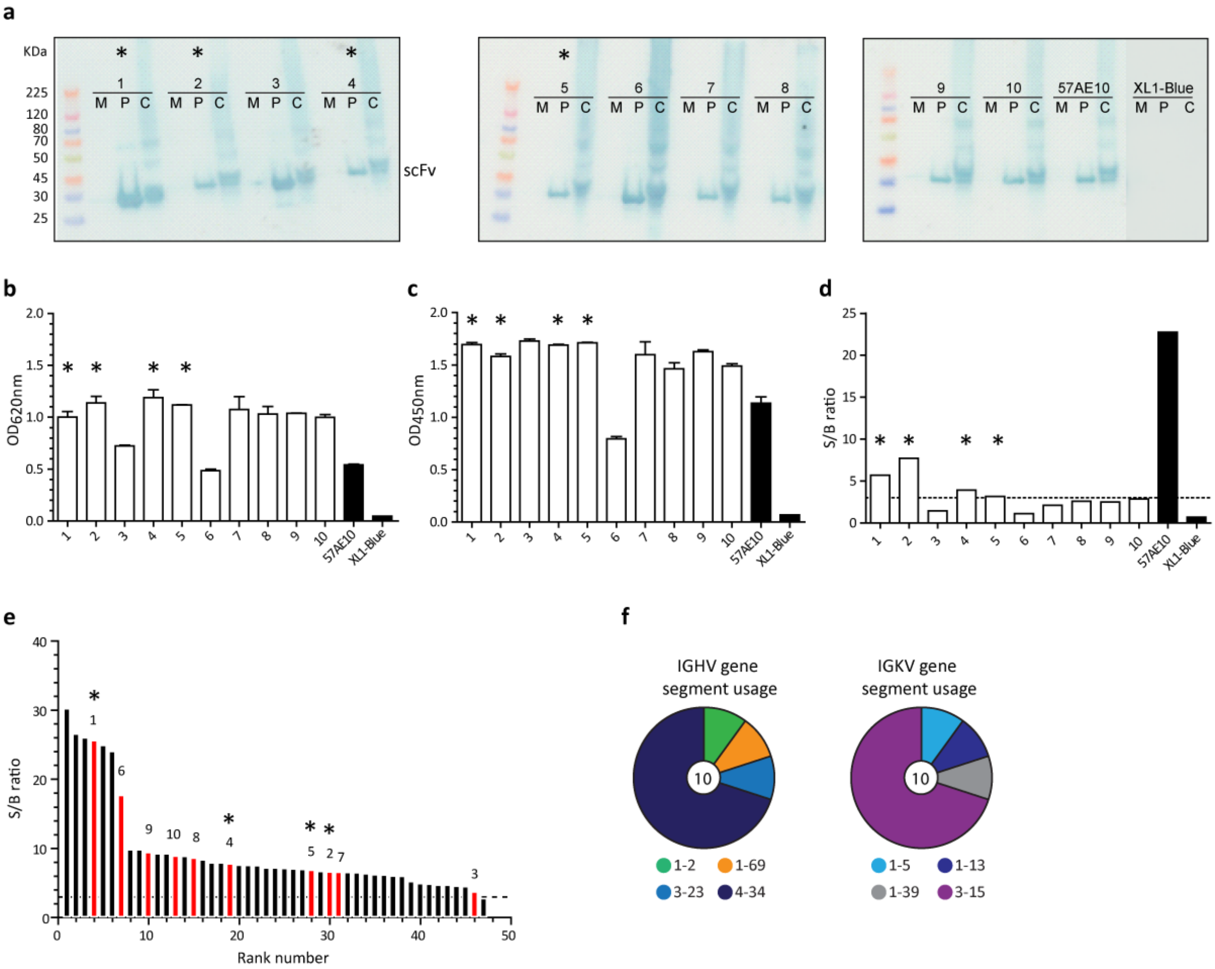

Supplementary Figure S4. Characterization of pIII HV clones. To characterize single clones from pIII HV, 10 clones classified as positive in the single-phage clone ELISA (Fig. 4b) were chosen. (**a**) The encoded scFvs, along with positive and negative controls, were sub-cloned into a vector for soluble expression and expressed in *E. coli* followed by sub-cellular fractionation into medium, periplasmic and cytoplasmic fractions (M, P, C) and Western blotted to detect expressed protein. (**b, c**) Periplasmic fractions were analyzed by (**b**) protein L ELISA and (**c**) anti-c-myc tag ELISA and confirmed to be functional and full-length scFv, respectively. (**d**) Antigen binding of scFv periplasmic fractions was assessed by OMV ELISA. A S/B ratio ≥3 is regarded as positive. (**e**) The graph is modified from Fig. 4b to illustrate the positions of the 10 selected clones (shown as red bars). The numbering shows the

clone numbers, those with * are the ones with positive binding in the OMV ELISA in d. (**f**) The 10 clones were sequenced and V gene usage identified.

Supplementary Figure S5

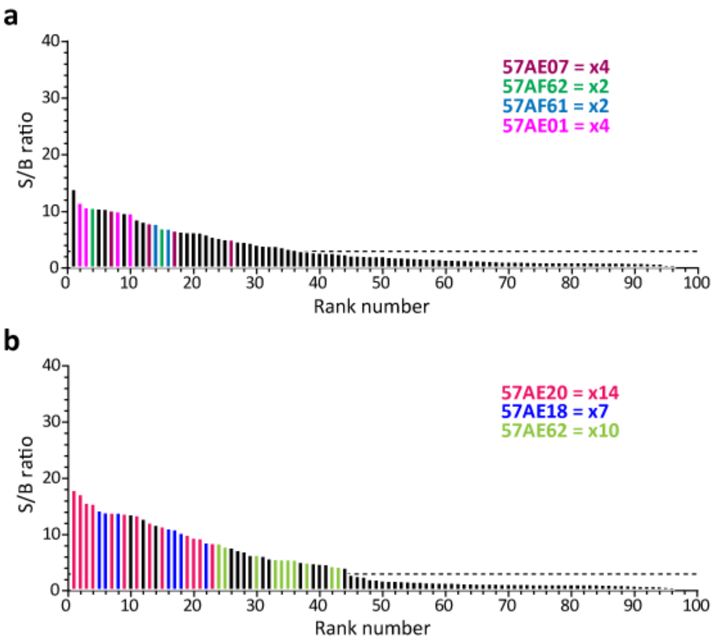

Supplementary Figure S5. Distribution of enriched clones in pIII LV and pIII HV after temperature challenge. Modified from Fig. 7c to illustrate positions of enriched clones which retained antigen binding after temperature challenge in (**a**) pIII LV and (**b**) pIX HV. Unique clones are identified by color and clone names from Supplementary Table S1. Black bars are either single clones or not sequence identified. Notably, all members of a clone either retained or lost binding.

Supplementary Figure S6

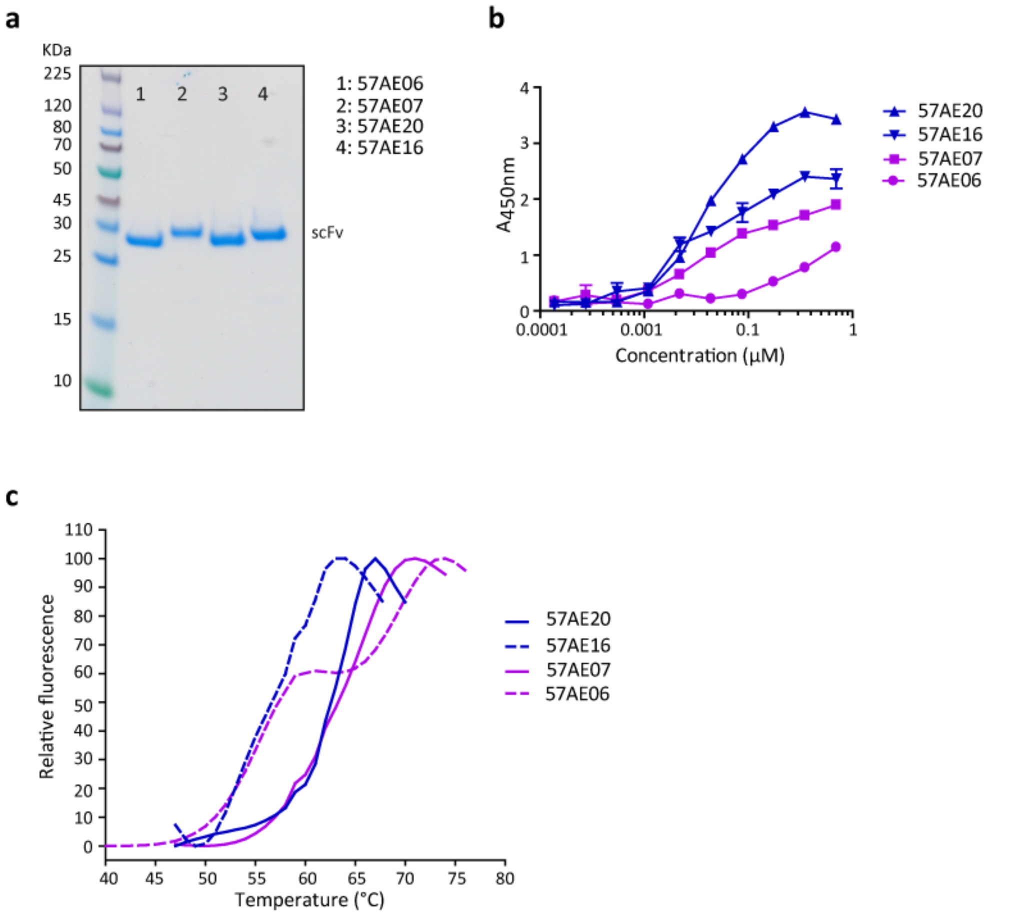

Supplementary Figure S6. Purification and validation of expressed scFvs. The highest and lowest ranking scFvs based on relative OMV binding strength (Fig. 7d) were expressed in *E. coli* and purified from periplasmic fractions by protein L affinity chromatography, followed by size exclusion chromatography. 57AE07 (highest) and 57AE06 (lowest) were chosen from pIII LV and 57AE16 (highest) and 57AE20 (lowest) were chosen from pIX HV. **(a)** Representative SDS-PAGE gel (n=2) showing a single band at approx. 30 KDa corresponding to the mass of the scFvs. **(b)** Representative ELISA showing OMV binding of normalized, titrated amounts of scFvs. Essentially the same result was obtained with separately expressed material; either protein L affinity purified scFv clones (n=3) or scFv clones purified by protein L followed by size exclusion chromatography (n=2). The results are given as mean ± SD of duplicates. pIX clones are shown in blue and pIII clones in magenta. **(c)**

Representative DSF melting curves (n=2-4). Samples were run in duplicates and the average signal is shown. scFv 57AE06 do not show a two-state transition curve, indicating a more complex unfolding.

Supplementary Table S1. Gene segment usage and enrichment of OMV-specific clones

**pIII LV**						
	**VH domain**			**VL domain**		
**Clone**	**IGHV**	**IGHD**	**IGHJ**	**IGKV**	**IGKJ**	**# of clones**
57AE00	4-34*01	3-22*01	4*02	3-15*01	5*01	1
57AE06	3-23*01	6-13*01	3*01	1-33*01	2*02	1[a]
57AE01	4-34*01	2-2*02	4*02	3-20*01	1*01	4
57AE02	4-34*01	3-10*01	4*02	1-27*01	3*01	1
57AE03	4-34*01	3-22*01	6*02	3-15*01	2*02	1
57AE05	1-69*12	2-2*02	4*02	3-20*01	2*01	1
57AE07	4-34*01	2-2*02	4*02	3-20*01	4*01	4
57AE48	3-21*01	5-12*01	4*02	3-15*01	2*01	1
57AE49	4-34*01	3-16*01	4*02	3-20*01	3*01	1
57AE52	3-48*04	1-1*01	4*02	1-39*01	1*01	1
57AE53	3-21*01	1-26*01	5*02	3-15*01	1*01	1
57AF51	1-3*01	5-12*01	4*02	1-33*01	3*01	1[b]
57AF52	1-3*01	5-12*01	4*02	1-33*01	5*01	1
57AF53	3-30*04	4-23*01	3*02	3-20*01	1*01	1
57AF54	3-21*04	6-13*01	3*02	3-20*01	2*02	1
57AF55	4-34*01	6-13*01	5*02	3-20*01	5*01	1
57AF56	4-34*01	5-24*01	6*02	3-20*01	1*01	1
57AF57	4-34*01	3-16*01	4*02	3-20*01	3*01	1
57AF59	4-34*01	2-2*01	6*02	3-20*01	5*01	1
57AF61	3-23*01	1-26*01	4*02	1-39*01	4*01	2[c]
57AF62	4-34*01	2-2*01	4*02	3-20*01	4*01	2
**pIII HV**						
57AE10	4-34*01	2-15*01	6*02	3-15*01	2*01	1[c]
57AE08	1-2*02	4-23*01	5*02	1-39*01	3*01	2
57AE09	3-23*01	6-13*01	3*01	1-33*01	2*02	3[a]
57AE11	3-23*04	1-26*01	3*02	1-39*01	4*01	3
57AE12	3-23*04	1-26*01	3*02	3-20*01	1*01	1
57AF65	1-3*01	5-12*01	4*02	1-33*01	1*01	2
57AF67	1-69*12	6-13*01	4*02	3-15*01	4*01	1
**pIX LV**						
57AE13	4-34*01	2-15*01	6*02	3-15*01	2*01	1[d]
57AE14	4-34*01	5-24*01	4*02	3-20*01	4*01	1
57AE59	4-34*01	3-22*01	4*02	3-15*01	5*01	2
57AE60	3-74*02	3-22*01	5*02	3-15*01	4*01	3
57AF70	2-5*01	3-16*02	2*01	3-15*01	1*01	1
57AF71	1-3*01	5-12*01	4*02	1-33*01	3*01	1[b]
57AF72	3-23*01	4-17*01	4*02	1-13*02	1*01	2
**pIX HV**						
57AE16	3-21*01	5-18*01	5*02	3-20*01	1*01	9
57AE17	3-23*04	1-7*01	4*02	3-20*01	4*01	5
57AE62	3-23*01	1-26*01	4*02	1-39*01	4*01	10[c]
57AE20	3-23*04	3-22*01	4*02	1-5*03	2*01	14
57AE22	3-48*03	4-17*01	5*02	1-33*01	5*01	2
57AE31	4-34*01	4-17*01	4*02	3-20*01	4*01	1
57AE18	4-34*01	3-3*01	6*02	3-20*01	2*02	7
57AF76	4-34*01	3-10*01	4*02	3-15*01	1*01	1
57AF77	3-23*04	5-18*01	5*01	1-9*01	2*01	1
57AF80	3-48*01	6-6*01	5*02	1-39*01	4*01	1

[a] Identical clone in pIII LV/pIII HV
[b] Identical clone in pIII LV/pIX LV
[c] Identical clone in pIII LV/pIX HV
[d] Identical clone in pIII HV/pIX LV
Red: clones which dropped below S/B ≥ 3 after a 10 min heat challenge at 55°C.

Supplementary Table S1. Gene segment usage of OMV-specific clones. Single clones with S/B ratio ≥3 (Fig. 4c) were sequenced and gene segment usage identified. Random positive clones were picked from pIII LV and pIX HV, while all were sequenced from pIII HV and pIX LV. Enrichment of clones is indicated by number of identical clones found. Clones marked in red lost antigen-binding after temperature challenge at 55°C for 10 min (Fig. 7c and Supplementary Fig. S5).
